# Assessment on the Current State of On-Farm Diversity and Genetic Erosion in Barley (*Hordeum vulgare* L.) Landraces from Bale Highlands, Southeast Ethiopia

**DOI:** 10.1155/2021/6677363

**Published:** 2021-02-23

**Authors:** Fekadu Gadissa, Meskerem Abebe, Berhane Worku

**Affiliations:** ^1^Department of Biology, Madda Walabu University, Box 247, Bale Robe, Ethiopia; ^2^Department of Statistics, Mada Walabu University, Box 247, Bale Robe, Ethiopia

## Abstract

Barley landraces is among the major cereal crops grown in Ethiopian highlands including Bale highlands. However, in recent days, the crop is highly declining to the extents of total loss. This study was, therefore, aimed at assessing the extents of its on-farm diversity and genetic erosion from Bale highlands, Ethiopia. Data were generated from twelve administrative districts and analyzed considering important ecological and genetic erosion models. A total of 25 distinct (at least in naming) barley landraces with varying distribution patterns have been identified in the areas. Landrace richness (*R*) revealed higher magnitude among all the study districts, the smallest being 2.02 (*D*_Mg_) and 1.41 (*D*_Mn_) and considerable range of variations (*D*_Mg_ = 2.02 to 5.02, *D*_Mn_ = 1.41 to 3.17). Among the study districts, Dinsho consisted the highest on-farm diversity estimate (*D*_Mg_ = 5.02, *D*_Mn_ = 3.17) followed by Goba and Sinana (*D*_Mg_ = 4.50 and 3.97; *D*_Mn_ = 2.87 and 2.57 in that order). Estimate of the landrace evenness (*E*) also showed the highest magnitude (>0.95) except in Agarfa district (0.77). The result suggests potentiality of the areas and wide cultivation of majority of the landraces in the villages. However, nowadays, only 14 landraces are under cultivation and the remaining 11 are totally eroded from the district(s) constituting the highest (56.0%) combined genetic erosion suggesting loss of important agronomic traits and, thus, a major bottleneck for further improvement and conservation plans. Thus, attention should be payed to conserving the landraces for better further use.

## 1. Introduction

Genetic erosion refers to loss of genetic variability over space and time [1, 2]. It could be detected at various levels of taxonomic units such as at a species, population, or biodiversity level as well as at different geographic ranges. In real sense, it represents either the loss of entire populations or the loss or change in frequency of specific alleles particularly, rare alleles or allele combinations present within a population or in a given species as a whole. It commonly occurs in native (indigenous) species and often caused by human-driven or -related activities. The term was publicly used to refer to the loss or replacement of primitive races and varieties usually called landraces in the case of cultivated plants. It has become a critical agenda for the international agricultural community since the mid-1900s [3].

One of the negative consequences of genetic erosion is that it increases susceptibility to biotic and abiotic stresses [4] and, hence, reduces evolutionary potential and reproductive fitness of a given population or species over space and time [5]. Thus, in order to avert the problem and for mitigating production bottlenecks and supporting food security especially in resource-poor countries, *in situ* conservation of genetic resources especially in areas of domestication or origin, where diversity of genetic resources is concentrated, is very essential [6, 7]. Likewise, maintaining on-farm genetic diversity and farmers' indigenous knowledge along with their behavioral practices of keeping landraces of ancestral crop populations are also another equally important strategy for conserving crop species [8, 9]. Keeping the landraces and/or reversing their loss is absolutely essential since they are potential sources of materials for modern and stable selection breeding and for developing lines that are resistant to biotic and abiotic stresses. Moreover, they are adaptable to marginal and more diverse agricultural environments because of their rewarding evolutionary and adaptive potentials to overcome the recent unpredictable climatic effects [10, 11].

However, on-farm genetic resource conservation and research activities targeting improvement of indigenous crops received less attention in several countries [12, 13]. Ethiopia is one of those countries regardless of being the world's rich biodiversity center and harboring a variety of distinct food crops. In recent days, the country is under severing threat of loss in genetic diversity and most of the indigenous food crops are at risk of total extinction [11, 14, 15]. Ethiopian barley (*Hordeum vulgare* L.) landraces are among those crops regardless of their valuable and distinct agronomic traits [16]. Their cultivation is declining from time to time and in recent decades, only practiced by smallholder farmers for subsistence use only [17–20]. Attempts made so far to conserve the crop is very less except few explorations and rescue collections targeting maintenance under *ex situ* conditions at the Ethiopian Biodiversity Institute (EBI), formerly established as the Plant Genetic Resources Centre of Ethiopia (PGRC/E) through the International Board on Plant Genetic Resources in 1974 (currently the International Plant Genetic Resources Institute (IPGRI)). If well managed, however, it will become important sources of breeding and improvement because of their unique and long-term adaptive and evolutionary potentials to different biotic and abiotic stresses.

Bale highlands are one of the potential agricultural areas in the country and are well known in higher diversity and wider cultivation of Ethiopian barley landraces. However, in recent days, both the diversity and cultivation are highly declining to the extents of total loss of some previously important landraces from areas where they had been widely cultivated and most are pushed to marginal growing conditions. The decline in diversity and production is attributed to several and interrelated factors such as widespread adoption of modern and exotic varieties, recent climate change that resulted in habitat destruction and recurrent drought, and advancements in agricultural technology including a shift towards using mechanized farming that totally favored crops like wheat that are largely produced in bulk for commercial and industrial purposes. In addition, lack of research activities targeting its breeding and improvement and lack of well-documented study on the extents of its genetic erosion has hampered its conservation and improvement programs [7, 21–23]. Therefore, the present study was initiated in view of documenting the diversity of barley landraces from Bale highlands, Southeast Ethiopia, through various diversity indices and genetic erosion models. The information generated would lay bases for conservation and breeding of the landraces.

## 2. Research Methodology

### 2.1. Description of the Study Area

**Figure 1:**
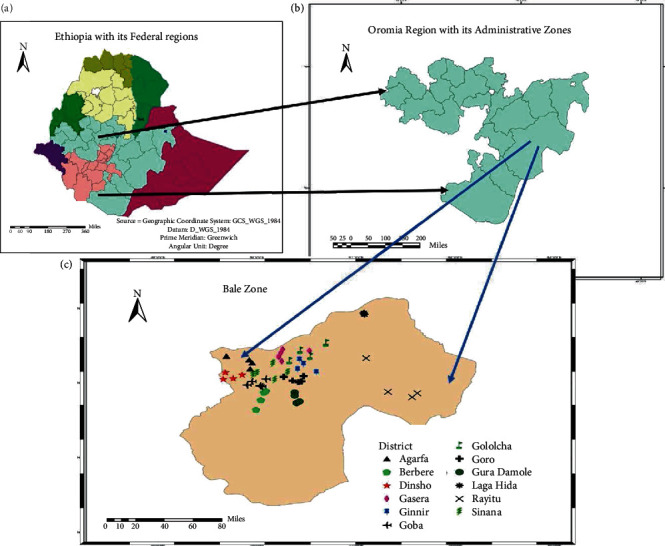
Map of Ethiopia with its Federal regions (a), Oromia regional state with its administrative zones (b), and Bale zone (c) showing the 12 districts covered during data collection mission.

The study was carried out in Bale zone, Southeast Ethiopia. Robe, the zonal city is 430 km from Addis Ababa, the capital of Ethiopia with 7° 08′ N (latitude), 39° 59′ E (latitude). The study encompassed 12 potential barley growing districts in the zone (Table 1; Figure 1). The districts were selected on the bases of their long-term (30 years and above) barley landraces growing practices and recent shift towards favoring wheat and less uniformity in sociocultural and religious outlooks of the farmers that posed some pressure on the selection of landraces for their end-use qualities.

### 2.2. Conceptual Approach

Until recently, one of the major bottlenecks in assessing the extents of on-farm genetic diversity and genetic erosion in a given crop species is lack of standardized and well-developed methodology. The problem is partly attributed to the relatively longer time required to gather adequate data, the multidisciplines required to end up with a concrete justification, and the dynamicity of genetic diversity itself in time and space [1, 24, 25]. In addition, access to sufficient traditional farmers-based scientific data on the selection, maintenance, and conservation of a given species is very limited [24, 26]. The problem is even worse in areas where traditional varieties, usually called landraces, are predominantly cultivated largely because of their localized maintenance and end-use qualities that have, to some extent, marginalized the crops being part of national and international research focus.

Ethiopia is among the countries facing the problem and has very scarce, if not none, detailed baseline information on the extents of on-farm genetic diversity and genetic erosion in indigenous food crops and their wild relatives [15]. Barley landraces are among the indigenous food crops with very scarce such information and lacks standard methodology. Thus, the present study targeted assessing the extents of on-farm genetic diversity and genetic erosion using local traditional knowledge and practices of farmers who are engaged in barley landrace farming over the past couple of decades.

The present study was conducted considering the concepts revealing a strong linkage between farmer's traditional knowledge and extents of on-farm genetic diversity (genetic erosion) and the large concordance between local (vernacular) names given to landraces and their genetic distinctiveness [27–30]. Accordingly, assuming landraces as distinct species, the models developed by Magurran and Hammer et al. [31, 32] were employed to determine extents of on-farm genetic diversity and genetic erosion in the landraces. The models were used as an indicator of variability based on all the necessary confirmations and assumptions that are meant to minimize the possible discordances between the names assigned to landraces and their genetic distinctiveness. Some of the assumptions were the self-pollinating nature of the crop that plays a critical role in maintaining the existing genetic integrity over long periods of time. Similarly, uniformity among larger members of the community in terms of ethnicity and socioeconomic and sociocultural background was considered an input to minimize the problem of naming the same landrace by different names. Uniformity in the farming system and cropping patterns was also considered an important factor to minimize giving the same name to landraces with different morphological and physiological characteristics.

### 2.3. Research Design and Sample Size Determination

A community based cross-sectional research design was employed focusing on selected farmers' districts and kebeles within a district that were identified after a rapid preliminary informal survey and discussions with the zonal and district agricultural bureau experts. The information was gathered from both primary and secondary sources on 2019/20.

Sample size was determined using a standard formula suggested by Freund and Williams [33] considering a 95% confidence level and an error margin of less than 10% as proposed by Dickson and Nyariki [34].

Accordingly,  *N* = 0.25∕*SE*^2^ = 400, where SE (standard error) = 2.5%, which means 0.025, SE^2^ (standard error square) = 0.000625, and *N* is the sample size.

The total sample was fairly distributed to the study districts assuming equal coverage to obtain a relatively balanced response and conclusion.

### 2.4. Data Collection Methods

Valuable data were collected through a questioner, household interview, and focus group discussions (FGD) using key informants and personal observations at barley fields.

Questionnaire method was used with the intention of setting information from a wide range of sources (respondents) regarding the indigenous knowledge and practices involved in barley landraces farming, management, conservation, and utilizations in the study areas. The questionnaire was written in English and translated into local languages such as ‘*Afan Oromo*' and ‘*Amharic*' and distributed to the selected 400 household heads. The household heads were purposively selected based on the preliminary survey and documents from district agricultural offices. In addition, all the required age groups and sexes including elder women household heads were intentionally involved to guarantee good coverage of the required diversity in indigenous knowledge.

Interview questions were used to substantiate the information generated through the questionnaires. In this regard, semistructured questions that address matters regarding the barley landraces currently or used to be cultivated, extents of their production relative to other cereal crops, production challenges, and major utilizations were presented. The key informants were carefully selected from the household heads of both sexes and different age groups involved in the questionnaire method based on their willingness and rich practical knowledge on barley production, conservation, and utilization in the areas.

Focal group discussions were carried out with selected barley growing elders and experts to complement the information obtained from individual farmers and to minimize missing data. The key informants involved were well-recognized elder farmers aged 50 or more and spent their entire lives in the localities and were engaged in barley farming and seed selection. Open group discussions regarding the reasons why barley landraces are left marginalized, main factors for the current decline in production of the landraces, and their general views regarding the benefits of the landraces were presented. Finally, after thorough discussion, consolidated ideas were noted.

Agricultural extension experts and development agents (DAs) at all the selected districts and Peasant Association levels, as well as experienced researchers at Sinana Agricultural Research Institute, the regional institution located in the study zone and mandated for research on barley and wheat, were consulted to cross-check whether the landraces identified by the local farmers were really landraces or improved varieties. Furthermore, secondary data from the Ethiopian Biodiversity Institute (EBI) and barley researchers were used to validate the landraces and screen the improved and exotic varieties released through the formal system.

### 2.5. Data Analysis

Descriptive statistics regarding sociodemographic characteristics of the respondents were analyzed using Minitab version 19. The extents of on-farm genetic diversity in the crop were analyzed by using the different ecological models that have been adapted to species diversity. Accordingly, Margalef's, Menhinick's, Shannon-Weaver, and Simpson's diversity (equivalent of Nei's diversity) indices were employed assuming the landraces as distinct species [31], and thus, their diversity was explained in terms of richness (the number of landraces) and evenness (how equally are they abundant).

Computationally, landrace richness (intervarietal diversity) (*R*) among the twelve districts was compared by using Margalef's (*D*_Mg_) and Menhinick's (*D*_Mn_) indices as follows:

(1) $$\begin{array}{c} D_{\text{Mg}}=\frac{L-1}{\ln R},D_{\text{Mg}}\geq 0,\\ D_{\text{Mg}}=\frac{L}{\sqrt{R}},D_{\text{Mn}}\geq 0. \end{array}$$

where *L* is the number of landraces in each study district and *R* designates the number of records for each landrace.

Similarly, landrace evenness (*E*) was determined as a measure of Shannon-Weaver information index (GDs) and was given by *E* = GDs/ln*L*, where *L* is the total number of landraces cited in each study district and GDs is a measure of Shannon-Weaver information index and given by

(2) $$\begin{array}{c} \text{GDs}=-\overset{n}{\underset{i=1}{\sum }}Pi\ln Pi, \end{array}$$

where *Pi* is the proportional abundance of the *i*^th^ landraces and given by (*ni*/*N*, where *ni* is the number of each record and *N* is the total number of records in each district). Its magnitude is greater or equal to 0.

Spatial diversity or abundance of the landraces was computed using Simpson's index (*D*) by taking into account the frequency of occurrence of each recorded farmer landrace (assuming as a distinct species) in the total districts. It is given by

(3) $$\begin{array}{c} \;D=1-\overset{n}{\underset{i=1}{\sum }}{Pi}^{2}, \end{array}$$

where *Pi*^2^ is the squared proportion of landrace *i* to the total records.

Extents of genetic erosion in the barley landraces for each district and combined over the study area were determined in terms of temporal diversity (rate of change over time) and it was calculated over a period of twenty to thirty years following [32] which is given by

(4) $$\begin{array}{c} \text{GE}\%=100\%-\text{GI}, \end{array}$$

where GE is the extents of genetic erosion and GI is the extents of genetic integrity and computed as

(5) $$\begin{array}{c} \text{GI}\%=\left({\text{N}}_{2}/{\text{N}}_{1}\right)\times 100, \end{array}$$

where *N*_2_ refers to number of landraces currently cultivated in the study area and *N*_1_ refers to the number of landraces used to be cultivated over the past twenty to thirty years.

## 3. Results and Discussion

### 3.1. Sociodemographic Characteristics of the Study Population and Its Implications

Summary of the respondent's sociodemographic characters was presented in *Supplementary Table*1. Accordingly, 326 or 81.5% of the total population were males and the remaining 74 or 18.5% were females, most of which are divorced and/or widowed. Such larger number of males over females in all the study districts and their respective kebeles implies the less involvement of women and dominance of males in agricultural practices in general and barley production in particular. It clearly shows that female heads are yet under cultural impositions that prohibited their active participation in owning farmlands and agricultural activities and even the revenue generated from the activities.

Most of the respondents (88.8%) used to grow barley for more than a couple of decades (greater than 40 years old) revealing their rich knowledge and behavioral practices in barley landrace production. Hence, it suggests appropriateness of the study population in providing sufficient and valuable information regarding the landraces under cultivation or used to be cultivated over the last couple of decades (20 to 30 years ago) along with the main challenges of production and main utilizations.

Larger proportion of the respondents (333 or 83.25%) had less than primary school education but they have rich indigenous knowledge-based agricultural practices and, thus, long been involved in barley landrace selection, conservation, and maintenance processes. Similarly, regardless of dominance in Oromo ethnic group that are Muslims, the ethnic and religious heterogeneity in the area has also played a great role in the selection and conservation of some landraces for their specific endue qualities.

### 3.2. Barley Landraces Named in the Study Area and Their Distinctive Features

Indigenous knowledge-based vernacular (common) names given to genetic resources is among the indicators of genetic distinctiveness, and names are usually assigned from the points of view of their distinct characteristic features, specific or special end-use qualities, or other at least locally important attributes [20, 27, 28]. Therefore, assessing and documenting such information is an important aspect of facilitating conservation and further utilizations. Accordingly, twenty-five distinct barley landraces have been identified in Bale zone, Ethiopia (Table 2). They are entirely distinct at least in name, if not genetically, from similar studies made so far in different parts of the country [20, 35, 36]. The landraces are distinct in their seed color, spike length, number of rows, stress tolerance, yield, end-use qualities, and other important agronomic traits. Some of the landraces are still under active cultivation with different degrees of coverage at different localities. However, some are already lost and left their only memories behind, and few are highly marginalized and on the verge of perishing. There have been similar reports indicating the rapid loss of previously important landraces from Northern [20, 36] and Central [35] Ethiopia.

According to the farmers consulted, different characteristic features of the landraces or associated features that the farmers thought important for identification and showing distinctiveness had been used for assigning the names. For example, Kasale, Muga, Bira Adi, and Bira Dima were named to give emphasis to their seed colors such as deep black, faded black, white, and reddish colors, in that order, and thus to distinguish them from other related landraces. Butuji, and Barasdad were given names to reveal the short and strong stem length that could resist lodging. Farasgama, Kinkicho, Mukura, and Kate were named from the points of view of short spike length. Aruso Bale, Aruso Balticha, and Aruso Limat were given a common name ‘Aruso' to show their suspected area of origin in ‘Arsi,' one of the administrative zones in Oromia region that is adjacent to the study area, Bale zone. The second names, Bale, Balticha, and Limat were given to avoid confusion and show their distinctiveness from each other. Haji Yune and Kabe were named after a well-known farmer called Haji Yune and Kabe who were pioneer in cultivating the landraces. End-use qualities were also used in naming the landraces that have distinctive and specific utilizations. For example; Senef Gebs were named to show the ease of dehulling while preparing ‘*Kolo*' (roasted barley grain). Similarly, Kubsa was named to indicate its high flour quality.

Some of the special attributes of the landraces were noted and accordingly, most of them had white seed color revealing their end-use qualities related preferences by the local community assuming that food and beverage products produced from white seeded barley bear appealing color. However, being white does not imply their prevailing nutritional content and yet no supporting report in Ethiopia or elsewhere in the world. Another special feature common to most of the landraces is that they are two rowed representing the intactness of those original and ancestral landraces as suggested by [37]. As a result, they appear to bear good and original adaptive potentials to the current unpredictable environmental stresses and thus favored by the local farmers; otherwise, most are relatively low yielding when compared to those four- or six-rowed landraces derived from two-rowed ancestors through mutation. Similarly, most of the landraces noted bear long spikes, which is of course, a feature of the two-rowed ancestral landraces and suggests the country being the center of origin and/or diversity for the crop. A considerable number of the landraces were revealed to be tolerant to environmental stresses such as drought, cold, and rarely, lodging. Such special attributes once again suggest significance of the landraces in bearing unique and important gene(s) for selection breeding especially in the current scenario of rapid climate change. Several landraces were well appreciated for their good flour and baking qualities though few have a very restricted utilization. For example, Senf Gebsis mainly used for *Kolo* or roasted barley grain, Kasale and Mugaare used for making*Tella*, local fermented beer or *Kenetto*, local nonfermented beverage. Shewayrga et al. [19] reported a similar result on sorghum (*Sorghum bicolor* L. (Moench)) landraces from Northeastern Ethiopia. Similarly, Tsegaye and Berg [38] noted a similar use in Ethiopian tetraploid wheat landraces from Eastern Shewa, Central Ethiopia.

### 3.3. Distribution Patterns of the Landraces

Distribution patterns of the 25 barley landraces recorded in the present study were presented under Tables 3 and 4. Accordingly, the distribution pattern varied across the districts considered and hence landraces popular in one district or villages within districts were rare in others and *vice versa*. On the other hand, some of the landraces, for example, Kasale, Senef Gebs, Wadago, and Samareta were commonly cited in all the study districts (the first two) except one (the second two) with different proportions. Haji Yune, Gomboba, Bira Adi, Aruso Bale, and Muga are also common to most of the study districts suggesting their wide use for preparing different cultural and religious dishes in Bale highlands. However, some of the landraces had a very restricted distribution because of their localized end-use qualities. For example, Muga is restricted to Dinsho district where it is largely used for making *Tella* (local fermented beer) and *Keneto*, local unfermented and nonalcoholic drink prepared during cultural and religious ceremonies. Similarly, Kinkicho, Mukura, and Kate are each restricted to only two of the districts suggesting their restricted end-use qualities such as *Shorba*or *Soup* (Kinkich), *Besso*, or a recipe prepared from fine ground flour (Mukura), and *Kita* or unleavened and thicker pancake (Kate) that are common to the districts. All the remaining landraces had distribution frequency of three to six districts. The trend suggests artificial selection pressure targeting their end-use qualities and purposeful maintenance of some landraces to use for the intended purpose. There have been similar reports from all the study districts and even from across the barley growing regions of the country [20, 35, 36].

In general, the distribution patterns observed is a result of selection pressures that are directly or indirectly related to exploiting and excelling local utilizations of the crop. The selection process largely targeted favoring those landraces that bear higher market demand and good flour quality to meet the demand of household consumption, religious, or cultural ceremonies. For example; Senef Gebs has more market demand and helpful in generating cash to several youngsters in the localities; Kasaleis maintained for its religious and cultural related end-use qualities; Samareta and Wadago bear good flour quality because of their white seed color that is favored by the local communities in making *Marka* (*Genfo*, local thick porridge), *Injera* (leavened thin pancake), *Kita* (unleavened thick pancake). Similarly, selection was being made towards favoring landraces with a relatively good yield, bearing higher resistance to environmental stress, and shorter length of maturity to supplement the food shortage gap in extreme highland and degraded areas where wheat production is not suitable. In this regard, Aruso Balticha and Kasale are preferred because of their preferred quality in resisting extreme climatic conditions. As a result, on-farm diversity of the landraces is narrowing, and a larger number of the landraces have been neglected and eventually eroded despite their important agronomic traits and contribution to further targeted breeding. Tadesse and Asres [20] reported a similar decreasing trend on barley landraces from Northwestern Ethiopia.

### 3.4. Estimates of On-Farm Genetic Diversity

#### 3.4.1. Genetic Richness, Spatial Diversity Pattern, and Evenness of the Landraces

Summary of the interlandrace diversity or landrace richness (*R*) computed in terms of Margalef's (*D*_Mg_) and Menhinick's (*D*_Mn_) indices is presented in Table 3. As compared to the result from the country's well-acknowledged landrace diversity [20], both indices revealed a higher magnitude of the landrace richness among all the study districts, the smallest being 2.02 (*D*_Mg_) and 1.41 (*D*_Mn_) in Gololcha and Gura Damole districts, respectively, and considerable range of variations (*D*_Mg_ = 2.02 in Gololcha and Gura Damole to 5.02 in Dinsho, *D*_Mn_ = 1.41 to 3.17 in the same districts). The result suggests potentiality of Bale highlands in harboring larger number of barley landraces in broad sense and important agronomic traits (associated genes) in narrow sense that could be used for further breeding and conservation actions.

With regard to the study districts considered, Dinsho consisted the highest on-farm diversity estimate (*D*_Mg_ = 5.02; *D*_Mn_ = 3.17) followed by Goba and Sinana districts (*D*_Mg_ = 4.50 and 3.97; *D*_Mn_ = 2.87 and 2.57 in that order). The result suggests that these districts largely relied on barley cultivation because of their larger topography that is highly rugged, degraded, and not suitable for cultivation of other cereal crops. Thus, one can imagine how important the landraces are in supporting the livelihood of subsistence farmers living in extreme conditions. Gura Damole and Gololcha districts each scored relatively lower diversity estimate (*D*_Mg_ = 2.02; *D*_Mn_ = 1.41) implying dominance of wheat production in the areas. However, such a high diversity index sometimes might not necessarily indicate the extent of genetic diversity and possible importance of the crop since some districts may cultivate only limited barley landrace but in a large land mass that could constitute a very low diversity index.

In general, as compared to the potential areas in the country, for example northwestern highlands (24 landraces) [20], central or west Shewa highlands (14 landraces) [35], and north eastern highlands (15 landraces) [36], the districts are rich and may be considered one of the centers of origin and diversity for barley genetic resource and, thus, important sources of barley selection and conservation. Therefore, special research and intervention actions targeting conservation and improvement of those landraces needs to be put into practice beyond awaking the local community in advancing their indigenous knowledge-based utilizations.

Extent of diversity estimate in terms of evenness index (*E*) (Table 3) also showed the highest magnitude (greater than 0.95) except in Agarfa district, which scored 0.77. Such highest evenness in the districts was attributed to the wide cultivation and thus abundance of majority of the landraces scored across the villages or kebeles (the lowest administrative structure). Thus, the landraces as well as the districts are important sources of barley genetic resource conservation. There has been report by Tadesse and Asres [20] suggesting greater evenness in northwestern parts of the country, one of the potential barley growing areas. The report, together with the present result, indicates potentiality of the country for barley breeding and conservation.

#### 3.4.2. Genetic Abundance of the Landraces

**Figure 2:**
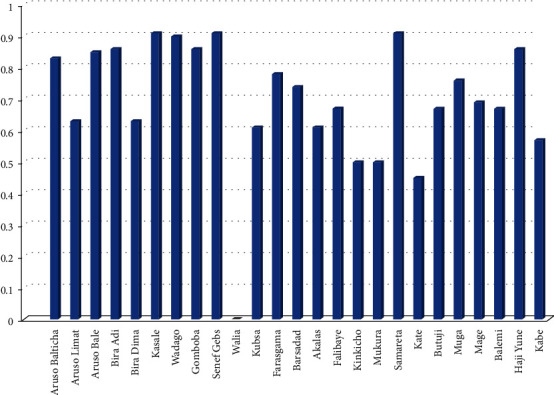
A bar chart showing genetic abundence of the 25 barey landraces expressed in terms of Simpson's diversity index (*D*).

Genetic abundance which was determined using Simpson's diversity index (*D*) considering occurrence of the landraces in one or more of the target locations is presented in Figure 2 and Table 4. Accordingly, nine landraces, namely, Samareta (0.91), Senef Gebs (0.91), Kasale (0.91), Wadago (0.90), Bira Adi (0.86), Haji Yune (0.86), Gomboba (0.86), Aruso Bale (0.85), and Aruso Balticha (0.83) in the order of magnitude, have scored a higher abundance. Likewise, nine landraces such as Farasgama (0.78), Muga (0.76), Barasdad (0.74), Mage (0.69), Balemi, Butuji and Falibaye (each 0.67), Aruso Limat (0.63), and Kubsa (0.61) showed moderate abundance. All the remaining landraces revealed a relatively lower abundance that ranged from 0.57 in Kabe to 0.00 in Walia and limited to only one or two collection districts. The patterns of genetic abundance suggest that those highly abundant landraces were under wide selection and cultivation because of their preferred end-use qualities or their improved potential in resisting environmental stresses. As a result, they may bear important agronomic traits that could be used for further intervention work. Likewise, the result may suggest the area as one of the sites to exploit barley genetic resources for conservation and breeding scheme and used as a clue for the country in general and the districts in particular to become among the areas of origin and domestication for barley genetic resource.

However, regardless of the abundance, some landraces are highly marginalized and unlike others, cultivated only in small quantities for their specific end-use qualities. For example, the black seeded landraces called Kasale and Muga are currently at high risk of extinction because of only their seed color that, according to farmers, does not seem appealing for making food items such as local *injera* (leavened thin pan cake type), *dabo* (local bread), and *Kolo* (roasted barley grain). In recent days, they are marginally cultivated on a very small plot of land mainly for making *Tela* (fermented local alcoholic beverage) and *Keneto* (local nonfermented beverage). Thus, special measures need to be considered in widening their cultivation and end-use qualities taking into account their nutritional contents before they completely erode.

#### 3.4.3. Temporal Genetic Diversity and Extents of Genetic Erosion in the Landraces

**Figure 3:**
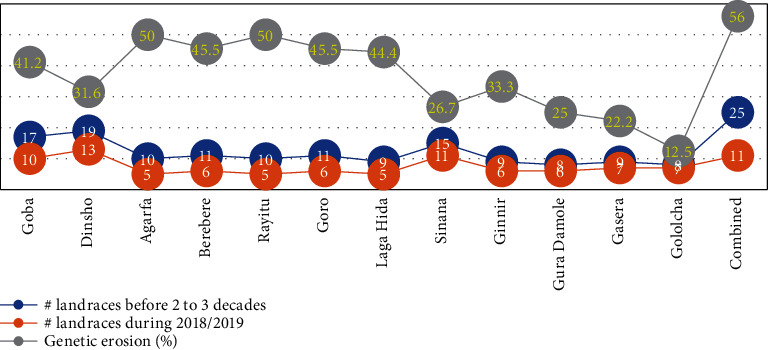
A chart showing temporal distribution of the 25 barley landraces along with the extents of genetic erosion in the 12 districts considered and when combined.

Temporal genetic diversity and extents of genetic erosion in the barley landraces considered from Bale highlands is presented in Figure 3 and Table 5. Accordingly, from the total of 25 barley landraces recalled, only 11 are grown at present time and the remaining 14 were totally eroded from the district(s) where they had previously been cultivated. As a result, estimate of their combined genetic integrity is lower (44.0%) with eventual extent of their combined genetic erosion being higher (56.0%). A similar increasing trend in the extents of genetic erosion on barley landraces have been reported from different parts of the country [15, 20]. With regard to the study districts, six, Gololcha (87.5%), Gasera (77.8%), GuraDamole (75%), Sinana (73.3%), Dinsho (68.4%), and Ginnir (66.7%), had moderately higher genetic integrity and consequently lower genetic erosion. The remaining districts, however, had considerably higher extents of genetic erosion with Rayitu and Agarfa districts (each scored 50%) being the highest.

The result suggests that though Bale highland is containing higher spatial barley genetic resources, the diversity is in a rapid shift towards declining integrity, and consequently, a larger number of genetic resources have been eroded and kept eroding through time. Such pronounced germplasm loss through time is resulting in loss of important agronomic traits and, thus, a major bottleneck for further improvement and conservation plans.

On the other hand, those integral landraces have continuously been cultivated and maintained because of their relatively high grain yield, unique end-use qualities, wide adaptation to the changing environmental conditions, local market price, and wide ethnobotanical uses. For example, Senef Gebs is maintained because of its significant contribution in generating cash to the youngsters as it is mainly used to prepare ‘*Kolo*' (roasted barley grain) which is sold in markets and along road sides. Bira Adi is mainly used as malt barley to brewery factories in the country and, thus, the main source of family income. Similarly, Aruso Balticha, Kasale, and Gomboba are preferred for their special end-use qualities in preparing foods and beverages for cultural and religious ceremonies.

Some of the landraces that have totally been lost had high abundance as indicated in Table 4. For example, Wadago, Haji Yune, Farsgama, and Barsadad scored an estimated abundance index of 0.90, 0.86, 0.78, and 0.74, respectively. This suggests that the landraces had widely been cultivated and were useful to the local community and the world at large. Thus, their erosion seems largely anthropogenic and attributed to intensive artificial selection processes practiced in demand of high yield or other locally preferred traits. Likewise, scarcity of research interventions to improve their yield, maturity length, and stress tolerance has contributed a lot. Otherwise, they were believed to have a better accumulation of adaptive potential particularly in the current unpredictable climatic change.

## 4. Conclusions

Farmers' varieties (landraces) are genetic stocks for improvement and maintenance. Ethiopia is one of the potential countries in harboring farmers' varieties including cultivated barley genetic resources. Bale highlands is among the potential areas in the country and could be the hotspots for the barley landrace conservation and breeding as evidenced from the present study that revealed a large number of (25) distinct landraces, at least in naming. Furthermore, the patterns of on-farm genetic diversity in the landraces and the study districts observed signal the rich genetic resource in the country and could assure the country's being one of the centers of origin and/or diversity for cultivated barley genetic resource since extents of genetic diversity is one of the indicators of areas of origin or diversity for a given genetic resource. However, in recent days, the landraces are declining both in terms of their cultivation and number (only fourteen landraces are under cultivation out of the total twenty-five). As a result, the landraces are suffering from extreme genetic erosion (56.0%) that eventually signposts a rapid loss of important agronomic traits. Likewise, specific end-use quality-based artificial selection pressure imposed by local farmers is marginalizing some of the landraces, for example, *Kasale*, and pushing them closer to total extinction. In general, in order to exploit the maximum benefits from the crop and to meet future food needs for the world's rapidly growing human population, policymakers and researchers should pay attention to on-farm conservation and enhancement of the farmers' varieties.

## Figures and Tables

**Table 1:** Sampled districts (woredas) along with their total number of respondents by gender and geographic positioning used in the present study.

Sampling woreda (population)	Number of respondents			Altitude (m.a.s.l)	GPS reading (DD)	
	Male	Female	Total		Latitude	Longitude
Sinana	27	7	34	2341-3000	7.04-7.26	39.82-40.33
Agarfa	29	5	34	1992-3114	7.19-7.37	39.57-40.06
Dinsho	28	6	34	2801-3354	7.06-7.14	39.68-39.88
Gura Damole	26	7	33	1741-2226	6.73-6.87	40.38-40.50
Berebere	28	5	33	1591-2103	6.64-6.89	39.99-40.09
Gasera	27	6	33	1745-2412	7.23-7.45	40.01-40.26
Rayitu	25	8	33	1264-1676	6.81–7.33	41.10–41.62
Ginnir	27	6	33	1888-2375	7.14-7.31	40.30-40.73
Goba	27	7	34	2434-3500	6.77-7.05	39.72-40.11
Gololcha	27	6	33	1907-2526	7.30-5.54	40.33-40.70
LagaHida	28	5	33	1669-1733	7.92–7.94	41.07–41.08
Goro	27	6	33	1750-2623	6.92-7.09	40.20-40.53
Total study population	326	74	400			

DD: decimal degree; m.a.s.l: meter above sea level.

**Table 2:** Summary of the barley landraces cited by household heads in the study districts, Bale zone, Ethiopia, along with their seed color, description of the naming's, number of rows, and some special attributes.

S/N	Landraces	Seed color	Description of the naming's	# rows	Special attributes
1	Aruso Balticha	White	To specify its origin in Arsi^∗^ and wide distribution in Bale	2	Short spike, tolerant to stress, good flour quality
2	Aruso Limat	White	Named after its origin in Arsi^∗^ and brought to Bale later	6	Long spikes, tolerant to drought, good flour quality
3	Aruso Bale	White	Suspected to be originated at both Arsi^∗^ and Bale	6	Long spike, resistant to drought and cold, good flour quality
4	Bira Adi	White	Shows its being white colored and use in making beer malt	2	Long spikes, susceptible to stress, good flour quality, long plant height
5	Bira Dima	Purplish red	Shows its being red colored and use in making beer malt	2	Long spikes, susceptible to drought and cold, long plant height
6	Kasale	Black	Named to show its deep black seed color	2 or 6	Have long spikes, resist to cold stress
7	Wadago	Purplish red	Named to qualify its high tolerance to stress	6	Short spikes, short plant height, tolerant to lodgings
8	Gomboba	White	Named to signify its long and bending spikelet	2	Long spike, long plant height, good flour quality
9	Senef Gebs	White/black	Named to mean ‘lazy barley' to qualify it is easy to dehull	2	Long spikes, low yield, mainly used for *Kolo* (roasted barley grain)
10	Walia	White/black	A name given to reflect its endemicity as Walia Ibex	6	Short spikes, short plant height, susceptible to drought and cold
11	Kubsa	White	The name given to reflect its good flour and dough quality	2 or 3	Long spike, long plant height, good flour quality
12	Farasgama	White	Named to show its short and stunted plant and spikelet length	2	Short spike, smaller seed size, short plant height, tolerant to lodging
13	Barsadad	White	To show its long-term endemicity	2	Longer spike, short plant height, tolerant to lodging
14	Akalas	White	Given to show its strength in withstanding lodging	2	Longer spike, short plant height, tolerant to lodging
15	Falibaye	Purple	Named to represent its high resistance to environmental stresses	3 or 4	Large spike, seed size, and plant height, good flour quality,
16	Kinkicho	White	Naming that signify its short plant height	2 or 4	Small spike, small seed size, easy to dehull, short plant height
17	Mukura	White	Given to reveal its stunted plant height	2	Short spike and plant height, small seed size, easy to dehull
18	Samareta	White/purplish	Named to reflect its very attractive whitish-purple seed color	2	Large spike, seed size, and plant height, susceptible to lodgings
19	Kate	White	Named to signify its seed resemblance to wheat	4	Short spike and plant height, tolerant to lodging
20	Butuji	White	Named to indicate its short and strong plant resistant to lodging	6	Short spike, short plant height, large grain size and tolerant to lodging
21	Muga	Blackish	Named to reflect its faded black seed color	6	Large spike, larger seeds, high grain yields and good flour quality
22	Mage	Whitish	Named to show its longer and thinner seeds	2	Large spike, tolerant to stresses, good flour quality
23	Balemi	White	Named to reflect its being spiky	4	Long spike, resistant to stress, preferable flour quality
24	Haji Yune	Whitish	Named after the person ‘'Haji Yune'	4	Long spike, tolerant to drought and cold, larger sized seeds
25	Kabe	White	Named after the person ‘Kabe'	2	Short spike, small sized seeds, tolerant to lodging

^∗^Arsi is one of the administrative zones in Oromia regional state, Ethiopia.

**Table 3:** Summary of the interlandrace genetic evenness (*E*) and diversity indices computed in terms of the Shannon-Weaver index (GDs) and Margalef's (*D*_Mg_) and Menhinick's (*D*_Mn_) models.

Study districts	Diversity indices					
	Number of landraces (*L*)	Number of records (*r*)	Shannon-Weaver index (GDs)	Landrace evenness (E)	Margalef's index (*D*_Mg_)	Menhinick's index (*D*_Mn_)
Sinana	15	34	2.58	0.95	3.97	2.57
Agarfa	10	33	2.68	0.77	2.57	1.74
Dinsho	19	36	2.79	0.95	5.02	3.17
Gura Damole	8	32	2.06	0.99	2.02	1.41
Berebere	11	33	2.29	0.96	2.86	1.91
Gasera	9	33	2.11	0.96	2.29	1.57
Rayitu	10	33	2.21	0.96	2.57	1.74
Ginnir	9	33	2.11	0.96	2.29	1.57
Goba	17	35	2.69	0.95	4.5	2.87
Gololcha	8	32	2.05	0.99	2.02	1.41
Laga Hida	9	33	2.14	0.97	2.29	1.57
Goro	11	33	2.27	0.95	2.86	1.91

**Table 4:** Summary of the genetic abundance of the landraces determined using Simpson's diversity index (*D*).

Landraces	Number of citations												Total record	Simpson index (*D*)
	Sinana	Agarfa	Dinsho	Gura Damole	Berebere	Gasera	Rayitu	Ginnir	Goba	Gololcha	Laga Hida	Goro		
Aruso Balticha	4	5	3	7	—	—	—	6	3	—	—	—	28	0.83
Aruso Limat	1	—	1	—	2	—	—	—	—	—	—	—	4	0.63
Aruso Bale	3	—	3	—	—	3	5	—	2	4	—	4	24	0.85
Bira Adi	2	3	1	4	—	3	—	5	2	3	—	—	23	0.86
Bira Dima	1	2	—	—	—	1	—	—	—	—	—	—	4	0.63
Kasale	4	4	3	6	5	4	4	7	4	6	5	5	57	0.91
Wadago	—	4	2	4	4	6	5	2	3	4	3	2	39	0.90
Gomboba	3	—	2	4	2	4	—	3	1	3	—	—	22	0.86
Senef Gebs	3	3	4	3	5	5	4	4	2	4	4	5	46	0.91
Walia	1	—	—	—	—	—	—	—	—	—	—	—	1	0.00
Kubsa	—	3	—	—	1	—	—	—	—	—	—	2	6	0.61
Farasgama	2	—	—	2	2	—	—	—	1	—	—	3	10	0.78
Barsadad	1	—	—	—	2	—	—	—	2	—	—	2	7	0.74
Akalas	—	3	—	—	—	—	—	2	1	—	—	—	6	0.61
Falibaye	—	—	2	—	—	—	2	—	2	—	—	—	6	0.67
Kinkicho	—	—	1	—	—	—	—	—	1	—	—	—	2	0.50
Mukura	—	—	1	—	—	—	—	—	1	—	—	—	2	0.50
Samareta	2	—	3	5	3	4	4	3	4	5	3	2	38	0.91
Kate	—	2	1	—	—	—	—	—	—	—	—	—	3	0.45
Butuji	2	—	2	—	—	—	—	—	—	—	2	—	6	0.67
Muga	3	—	1	—	3	—	—	—	—	3	—	1	11	0.76
Mage	1	—	1	—	—	—	2	—	—	—	3	—	7	0.69
Balemi	—	—	2	—	—	—	—	—	2	—	—	2	6	0.67
Haji Yune	—	2	2	—	4	3	3	—	3	—	3	5	25	0.86
Kabe	—	—	—	—	—	—	—	2	1	—	4	—	7	0.57

**Table 5:** Estimate of genetic erosion in barley landraces over the last 2 to 3 decades in each study district and combined over the districts.

Study districts	List of landraces lost over the past two to three decades							# landraces before 2 to 3 decades	# landraces during 2018/2019	Genetic integrity (%)	Genetic erosion (%)
Goba	Kabe	Farasgama	Barsadad	Mukura	Falibaye	Akalas	Haji Yune	17	10	58.8	41.2
Dinsho	Mukura	Kate	Mage	Haji Yune	Wadago	Falibaye		19	13	68.4	31.6
Agarfa	Kubsa	Kate	Akalas	Kate	Haji Yune			10	5	50.0	50.0
Berebere	Kubsa	Farasgama	Barsadad	Haji Yune	Wadago			11	6	54.5	45.5
Rayitu	Akalas	Falibaye	Mage	Wadago	Haji Yune			10	5	50.0	50.0
Goro	Kubsa	Farsgama	Barsadad	Haji Yune	Wadago			11	6	54.5	45.5
Laga Hida	Mage	Haji Yune	Kabe	Wadago				9	5	55.6	44.4
Sinana	Farasgama	Barsadad	Mage					15	11	73.3	26.7
Ginnir	Akalas	Kabe	Wadago					9	6	66.7	33.3
Gura Damole	Farasgama	Wadago						8	6	75.0	25.0
Gasera	Haji Yune	Wadago						9	7	77.8	22.2
Gololcha	Wadago							8	7	87.5	12.5
Combined								25	11	44.0	56.0

## Data Availability

The datasets generated during and/or analyzed during the current study are available from the corresponding author on reasonable request. Some are included in the supplementary information files.

## Supplementary Materials

Supplementary Materials Supplementary Table 1: demographic data of the respondents in each study district.
