# Supplementary material for: Assessment on the Current State of On-Farm Diversity and Genetic Erosion in Barley (Hordeum vulgare L.) Landraces from Bale Highlands, Southeast Ethiopia
Source: Biomed Res Int. 2021 Feb 23;2021:6677363. doi: 10.1155/2021/6677363 (PMC7929664; doi:10.1155/2021/6677363)
Supplement: Supplementary Materials — Supplementary Table 1: demographic data of the respondents in each study district. [file 6677363.f1.doc]

| Study variable | | | Sex | | | | Age | | | | | | Education Level | | | | | | | | Marital Status | | | | | | | | | Religion | | | | | | Ethnicity | | | | | | | |  |
| --- | --- | --- | --- | --- | --- | --- | --- | --- | --- | --- | --- | --- | --- | --- | --- | --- | --- | --- | --- | --- | --- | --- | --- | --- | --- | --- | --- | --- | --- | --- | --- | --- | --- | --- | --- | --- | --- | --- | --- | --- | --- | --- | --- | --- |
| Category | | | M | | F | | <= 25 | | 26-30 | | 31-40 | > 40 | Only read and write | Primary School | High school | | College Diploma | | Degree | | | Single | | Married | | Divorced | | | Widowed | | Muslim | Christian | | | Others | | | Oromo | | Amhara | | Others | | |
| No and proportion of Respondents at each study District | Dinsho | 28.0 | | 6.0 | | 0.0 | | 1.0 | | 3.0 | | 30.0 | 25.0 | 7.0 | | 2.0 | | 0.0 | | 0.0 | | | 0.0 | | 33.0 | | 0.0 | 1.0 | | | 27.0 | | 6.0 | 1.0 | | | 30.0 | | 4.0 | | 0.0 | |  | |
|  | % | 82.4 | | 17.6 | | 0.0 | | 2.9 | | 8.9 | | 88.2 | 73.5 | 20.6 | | 5.9 | | 0.0 | | 0.0 | | | 0.0 | | 97.1 | | 0.0 | 2.9 | | | 79.4 | | 17.7 | 2.9 | | | 88.2 | | 11.8 | | 0.0 | |  | |
|  | Sinana | 27.0 | | 7.0 | | 1.0 | | 1.0 | | 4.0 | | 28.0 | 23.0 | 9.0 | | 1.0 | | 1.0 | | 0.0 | | | 0.0 | | 34.0 | | 0.0 | 0.0 | | | 30.0 | | 4.0 | 0.0 | | | 29.0 | | 4.0 | | 1.0 | |  | |
|  | % | 79.4 | | 20.6 | | 2.9 | | 2.9 | | 11.8 | | 82.4 | 67.6 | 26.5 | | 2.9 | | 2.9 | | 0.0 | | | 0.0 | | 100.0 | | 0.0 | 0.0 | | | 88.2 | | 11.8 | 0.0 | | | 85.3 | | 11.8 | | 2.9 | |  | |
|  | Agarfa | 29.0 | | 5.0 | | 0.0 | | 1.0 | | 2.0 | | 31.0 | 28.0 | 5.0 | | 1.0 | | 0.0 | | 0.0 | | | 1.0 | | 31.0 | | 0.0 | 2.0 | | | 28.0 | | 6.0 | 0.0 | | | 27.0 | | 6.0 | | 1.0 | |  | |
|  | % | 85.3 | | 14.7 | | 0.0 | | 2.9 | | 5.9 | | 91.2 | 82.4 | 14.7 | | 2.9 | | 0.0 | | 0.0 | | | 2.9 | | 91.2 | | 0.0 | 5.9 | | | 82.4 | | 17.6 | 0.0 | | | 79.4 | | 17.6 | | 3.0 | |  | |
|  | Goba | 27.0 | | 7.0 | | 0.0 | | 0.0 | | 1.0 | | 33.0 | 25.0 | 6.0 | | 2.0 | | 1.0 | | 0.0 | | | 0.0 | | 33.0 | | 1.0 | 0.0 | | | 25.0 | | 8.0 | 1.0 | | | 28.0 | | 6.0 | | 0.0 | |  | |
|  | % | 79.4 | | 20.6 | | 0.0 | | 0.0 | | 2.9 | | 97.1 | 73.5 | 17.6 | | 5.9 | | 2.9 | | 0.0 | | | 0.0 | | 97.1 | | 2.9 | 0.0 | | | 73.5 | | 23.5 | 3.0 | | | 82.4 | | 17.6 | | 0.0 | |  | |
|  | Gura Damole | 26.0 | | 7.0 | | 1.0 | | 1.0 | | 3.0 | | 28.0 | 20.0 | 9.0 | | 3.0 | | 1.0 | | 0.0 | | | 1.0 | | 29.0 | | 2.0 | 1.0 | | | 24.0 | | 9.0 | 0.0 | | | 30.0 | | 3.0 | | 0.0 | |  | |
|  | % | 78.8 | | 21.2 | | 3.0 | | 3.0 | | 9.2 | | 84.8 | 60.6 | 27.3 | | 9.1 | | 3.0 | | 0.0 | | | 3.0 | | 87.9 | | 6.1 | 3.0 | | | 72.7 | | 27.3 | 0.0 | | | 90.9 | | 9.1 | | 0.0 | |  | |
|  | Berebere | 28.0 | | 5.0 | | 1.0 | | 1.0 | | 2.0 | | 29.0 | 26.0 | 5.0 | | 2.0 | | 0.0 | | 0.0 | | | 0.0 | | 33.0 | | 0.0 | 0.0 | | | 20.0 | | 10.0 | 3.0 | | | 28.0 | | 4.0 | | 1.0 | |  | |
|  | % | 84.8 | | 15.2 | | 3.0 | | 3.0 | | 6.1 | | 87.9 | 78.8 | 15.1 | | 6.1 | | 0.0 | | 0.0 | | | 0.0 | | 100.0 | | 0.0 | 0.0 | | | 60.6 | | 30.3 | 9.1 | | | 84.8 | | 12.2 | | 3.0 | |  | |
|  | Gasera | 27.0 | | 6.0 | | 0.0 | | 0.0 | | 3.0 | | 30.0 | 25.0 | 7.0 | | 1.0 | | 0.0 | | 0.0 | | | 0.0 | | 32.0 | | 1.0 | 0.0 | | | 29.0 | | 4.0 | 0.0 | | | 30.0 | | 2.0 | | 1.0 | |  | |
|  | % | 81.8 | | 18.2 | | 0.0 | | 0.0 | | 9.1 | | 90.9 | 75.8 | 21.2 | | 3.0 | | 0.0 | | 0.0 | | | 0.0 | | 97.0 | | 3.0 | 0.0 | | | 87.9 | | 12.1 | 0.0 | | | 90.9 | | 6.1 | | 3.0 | |  | |
|  | Rayitu | 25.0 | | 8.0 | | 0.0 | | 0.0 | | 1.0 | | 32.0 | 23.0 | 6.0 | | 3.0 | | 1.0 | | 0.0 | | | 0.0 | | 30.0 | | 2.0 | 1.0 | | | 27.0 | | 6.0 | 0.0 | | | 29.0 | | 2.0 | | 2.0 | |  | |
|  | % | 75.8 | | 24.2 | | 0.0 | | 0.0 | | 3.0 | | 97.0 | 69.7 | 18.2 | | 9.1 | | 3.0 | | 0.0 | | | 0.0 | | 90.9 | | 6.1 | 3.0 | | | 81.8 | | 18.2 | 0.0 | | | 87.8 | | 6.1 | | 6.1 | |  | |
|  | Ginnir | 27.0 | | 6.0 | | 1.0 | | 2.0 | | 3.0 | | 27.0 | 22.0 | 7.0 | | 2.0 | | 1.0 | | 1.0 | | | 1.0 | | 29.0 | | 1.0 | 2.0 | | | 28.0 | | 5.0 | 0.0 | | | 26.0 | | 5.0 | | 2.0 | |  | |
|  | % | 81.8 | | 18.2 | | 3.0 | | 6.1 | | 9.1 | | 81.8 | 66.7 | 21.2 | | 6.1 | | 3.0 | | 3.0 | | | 3.0 | | 87.9 | | 3.0 | 6.1 | | | 84.8 | | 15.2 | 0.0 | | | 78.8 | | 15.2 | | 6.0 | |  | |
|  | Gololcha | 27.0 | | 6.0 | | 0.0 | | 1.0 | | 2.0 | | 30.0 | 29.0 | 4.0 | | 0.0 | | 0.0 | | 0.0 | | | 0.0 | | 33.0 | | 0.0 | 0.0 | | | 24.0 | | 9.0 | 0.0 | | | 31.0 | | 2.0 | | 0.0 | |  | |
|  | % | 81.8 | | 18.2 | | 0.0 | | 3.0 | | 6.1 | | 90.9 | 87.9 | 12.1 | |  | | 0.0 | | 0.0 | | | 0.0 | | 100.0 | | 0.0 | 0.0 | | | 72.7 | | 27.3 | 0.0 | | | 93.9 | | 6.1 | | 0.0 | |  | |
|  | Laga Hida | 28.0 | | 5.0 | | 0.0 | | 2.0 | | 3.0 | | 28.0 | 25.0 | 7.0 | | 1.0 | | 0.0 | | 0.0 | | | 0.0 | | 28.0 | | 2.0 | 3.0 | | | 22.0 | | 11.0 | 0.0 | | | 29.0 | | 1.0 | | 3.0 | |  | |
|  | % | 84.8 | | 15.2 | | 0.0 | | 6.1 | | 9.1 | | 84.8 | 75.8 | 21.2 | | 3.0 | | 0.0 | | 0.0 | | | 0.0 | | 84.8 | | 6.1 | 9.1 | | | 66.7 | | 33.3 | 0.0 | | | 87.9 | | 3.0 | | 9.1 | |  | |
|  | Goro | 27.0 | | 6.0 | | 1.0 | | 1.0 | | 2.0 | | 29.0 | 26.0 | 6.0 | | 0.0 | | 1.0 | | 0.0 | | | 1.0 | | 30.0 | | 2.0 | 0.0 | | | 26.0 | | 7.0 | 0.0 | | | 25.0 | | 7.0 | | 1.0 | |  | |
|  | % | 81.8 | | 18.2 | | 3.0 | | 3.0 | | 6.1 | | 87.9 | 78.8 | 18.2 | | 0.0 | | 3.0 | | 3.0 | | | 3.0 | | 90.9 | | 6.1 | 0.0 | | | 78.8 | | 21.2 | 0.0 | | | 75.8 | | 21.2 | | 3.0 | |  | |
